# Global expression profile of tumor stem-like cells isolated from MMQ rat prolactinoma cell

**DOI:** 10.1186/s12935-017-0390-1

**Published:** 2017-01-31

**Authors:** Zhipeng Su, Lin Cai, Jianglong Lu, Chuzhong Li, Songbai Gui, Chunhui Liu, Chengde Wang, Qun Li, Qichuan Zhuge, Yazhuo Zhang

**Affiliations:** 1 Beijing Neurosurgical Institute, Capital Medical University, Beijing, 100050 China; 2 Department of Neurosurgery, First Affiliated Hospital of Wenzhou Medical University, Wenzhou, 325000 China; 3 Department of Neurosurgery, Beijing Tiantan Hospital, Capital Medical University, Beijing, 100050 China; 4 Beijing Institute for Brain Disorders Brain Tumor Center, Beijing, 100050 China; 5 China National Clinical Research Center for Neurological Diseases, Beijing, 100050 China

**Keywords:** Tumor stem-like cell, MMQ cell, Prolactinoma, VEGFA, Microarray

## Abstract

**Background:**

Cancer stem cells (CSCs), which have been isolated from various malignancies, were closely correlated with the occurrence, progression, metastasis and recurrence of the malignant cancer. Little is known about the tumor stem-like cells (TSLCs) isolated from benign tumors. Here we want to explore the global expression profile of RNA of tumor stem-like cells isolated from MMQ rat prolactinoma cells.

**Methods:**

In this study, total RNA was extracted from MMQ cells and MMQ tumor stem-like cells. RNA expression profiles were determined by Agilent Rat 8 × 60 K Microarray. Then we used the qRT-PCR to test the result of Microarray, and found VEGFA had a distinct pattern of expression in MMQ tumor stem-like cells. Then WB and ELISA were used to confirm the VEGFA protein level of tumor sphere cultured from both MMQ cell and human prolactinoma cell. Finally, CCK-8 was used to evaluate the reaction of MMQ tumor stem-like cells to small interfering RNAs intervention and bevacizumab treatment.

**Result:**

The results of Microarray showed that 566 known RNA were over-expressed and 532 known RNA were low-expressed in the MMQ tumor stem-like cells. These genes were mainly involved in 15 different signaling pathways. In pathway in cancer and cell cycle, Bcl2, VEGFA, PTEN, Jun, Fos, APC2 were up-regulated and Ccna2, Cdc25a, Mcm3, Mcm6, Ccnb2, Mcm5, Cdk1, Gadd45a, Myc were down-regulated in the MMQ tumor stem-like cells. The expression of VEGFA were high in tumor spheres cultured from both MMQ cell and human prolactinomas. Down-regulation of VEGFA by small interfering RNAs partially decreased cell viability of MMQ tumor stem-like cells in vitro. Bevacizumab partially suppressed the proliferation of MMQ tumor stem-like cells.

**Conclusions:**

Our findings characterize the pattern of RNA expression of tumor stem-like cells isolated from MMQ cells. VEGFA may act as a potential therapeutic target for tumor stem-like cells of prolactinomas.

**Electronic supplementary material:**

The online version of this article (doi:10.1186/s12935-017-0390-1) contains supplementary material, which is available to authorized users.

## Background

Prolactinomas are the most common subtype of pituitary adenomas with the proportion ranging from 40 to 66%, and occur with the population prevalence of 6–10 per 100,000 per year [1–3]. Since the mid–1980s, dopamine agonists (DA) have become the first-line therapy for the patients with prolactinomas [2, 4, 5]. However, it is not known how prolactinomas develop and tumor recurrence after withdrawal of DA treatment.

Cancer stem cells (CSCs) are defined as the unique subpopulation in the tumor, which possess the ability to initiate tumor growth and sustain self-renewal as well as metastatic potential [6, 7]. The further research discovered that cancer stem cells, which had been isolated from leucocythemia and other various malignancies, were closely correlated with the occurrence, progression, metastasis and recurrence of the malignant cancer [8–10]. However, little is known about the tumor stem-like cells (TSLCs) isolated from benign tumors. Xu [11] had proved for first time that tumor stem-like cells can be isolated from pituitary adenoma. Recently, Chen [12] and Mertens [13] had further demonstrated that the tumor stem-like cells were existed and identificated in pituitary adenoma. However, the global expression profile of RNA in tumor stem-like cells (TSLCs) isolated from prolactinoma kept unknown. Medical treatment is the first choice for prolactinoma [2, 4, 5], which results in limited tumor specimen source, moreover, there are small number of cells available from human prolactinoma postsurgical samples for in vitro experiments. Human pituitary adenomas cells grow very slowly in vitro [13], then it is difficult to get enough tumor cells to evaluate the molecular characteristic of TSLCs from human prolactinoma. MMQ rat prolactinoma cells are traditionally used as a substitutes for human prolactin-secreting pituitary adenoma cells in laboratory experiments, mainly because stably express prolactin (PRL) and have similar biological characteristics [14]. Therefore, research on characterizing RNAs expression of MMQ tumor stem-like cells will help to lay the foundation for human prolactinoma.

In this study, total RNA was extracted from three classical cultured MMQ cells and three MMQ tumor stem-like cells cultured in serum-free suspension medium containing growth factor. Then, their RNA expression profiles were determined by Agilent Rat 8 × 60 K Microarray. We validated these differentially expressed RNA by qRT-PCR and analyzed their potential function. Furthermore, the expression of VEGFA which over-expressed in the MMQ tumor stem-like cells was detected in tumor spheres isolated from human prolactinomas. Finally, we evaluated the cell viability after down-regulation of VEGFA by small interfering RNAs and the reaction to bevacizumab in MMQ tumor stem-like cells.

## Methods

### MMQ cell culture and reagents

MMQ rat prolactinoma cell line was used in this study, which was purchased from the Cell Bank of the Shanghai Branch of Chinese Academy of Sciences, and cultured in F12 medium (Gibco, Grand Island, NY, USA) supplemented with 2.5% fetal bovine serum (FBS, Gibco), 15% horse serum (Gibco) and 100 U/ml penicillin/streptomycin (Gibco). Cells were maintained in humidified atmosphere with 37 °C and 5% CO_2_.

### MMQ tumor stem-like cell culture and reagents

To obtain MMQ tumor stem-like cells, as described in our previous report [15], in brief, MMQ cells were cultured in DMEM/F12 medium (Gibco) supplemented with B27 (1×, Gibco), EGF (20 ng/ml, Preprotech, Rocky Hill, NJ, USA), bFGF (20 ng/ml, Preprotech) and 100 U/ml penicillin/streptomycin (Gibco). After been cultured for 2–3 weeks, MMQ tumor spheres can grow and form in serum-free suspension medium. Then, the MMQ tumor spheres were identified by immunofluorescence examination of stem cell markers and transplantation assay (see Additional file 1: Figure S1). Finally, the MMQ tumor spheres were resuspended and incubated with Rabbit anti-CD133 (1:50, MyBioSource, USA) for 30 min at 4 °C. After washing with PBS, cells were incubated with FITC-conjugated Donkey anti-Rabbit IgG (1:50, Santa Cruz, USA) for 30 min at 4 °C. Subsequently, FACS Calibur Flow Cytometer (BD Biosciences) was used to isolate and collect the CD133 positive tumor stem-like cells from the MMQ tumor spheres.

### Patients and tissue samples

Tumor specimens were obtained from pituitary prolactinomas resected via the transsphenoidal approaches in Department of Neurosurgery, First Affiliated Hospital of Wenzhou Medical University from March 2012 to December 2015. Research was approved by Clinical Medicine ethics Committee of First Affiliated Hospital of Wenzhou Medical University (permission: 2012–2013). Five pituitary adenomas samples (n = 5; 3 men and 2 women) were classified as prolactinomas according to the 2004 edition of the World Health Organization classification of pituitary tumors [16], which were ranged in age from 27 to 54 years. All had increased prolactin serum levels from 112.1 to >2000 ng/ml and the tumor volumes varied from 365 to 7800 mm^3^. Tumor tissues were immersed in DMEM medium (Gibco) for cell culture development.

### Human prolactinoma cell culture

Tissue samples were disaggregated with scalpel, washed in DMEM and fractured further by pipetting. Tumor cells were released from the tissues by enzymatic treatment with trypsase (Gibco) and grown in DMEM with 15% Fetal Bovine Serum (Gibco) until reaching confluency. Then tumor cells were collected and cultured in serum-free suspension medium which similar to MMQ tumor stem-like cells. Tumor spheres can grow and form in serum-free suspension medium after 2 to 3 weeks. Then, the human prolactinoma spheres were identified by immunofluorescence examination of stem cell markers (see Additional file 2: Figure S2).

### RNA extraction and evaluation

MMQ cells and CD133 positive tumor stem-like cells were homogenized in TRIzol reagent and total RNA was isolated according to the manufacturer’s protocol (Invitrogen, CA). The concentration and purity of total RNA were determined by an ultraviolet spectrophotometer at 260 and 280 nm.

### Microarrays and gene expression analysis

An Agilent gene expression array (KangChen Bio-tech Inc.) was used to investigate the global expression profile of MMQ tumor stem-like cells; the array represented more than 41,000 transcripts (http://www.kangchen.com.cn). The microarray datasets were normalized in Gene Spring GX using the Agilent FE one-color scenario (mainly quantile normalization). Differentially expressed genes were identified through fold-change screening (fold change ≥2.0). The gene ontology (GO) biological process and Kyoto encyclopedia of genes and genomes (KEGG) pathway enrichment analysis were performed using the Database for Annotation, Visualization and Integrated Discovery 6.7, (DAVID; http://david.abcc.ncifcrf.gov/) and were ranked by P values [17, 18].

### Verification of the RNA expression by qRT-PCR

Quantitative real-time RT-PCR (qRT-PCR) was used to verify RNA expression identified by Agilent Rat 8 × 60 K Microarray. The primers for the identified RNA were designed using Premier 5.0. Mixtures of 1 µg of total RNAs, 50 nM reverse primer, 5 units of M-MLV reverse transcriptase (Toyobo), 2 units of the RNAase inhibitor (Toyobo, Osaka, Japan) and 0.5 µM dNTP were incubated for 30 min at 16 °C, 30 min at 42 °C and 15 min at 70 °C. The reaction mixes were used as the templates for qRT-PCR using SYBR^®^ Green Real-time PCR Master Mix-Plus (Toyobo, Osaka, Japan) on the Applied Biosystems 7500 detection system. The PCR mixture including 1 µl RT product of total RNA, 10 µl SYBR-Green Real-time PCR Master Mix-plus, 2 µl plus solution, 2 µl each specific forward and reverse primers, and 3 µl DEPC water was made up the final volume to 20 µl. The reaction was performed at 95 °C for 2 min, followed by 40 cycles at 95 °C for 15 s and 60 °C for 1 min. Each sample was ran in triplicate and actin was used as internal control. Melting curves were used for verifying the specificity of each PCR reaction. The cycle threshold (Ct) was the mean value of three Ct values. The relative expression level of RNA was analyzed by the 2^−∆∆Ct^ method.

### PCR primers used were as follows

Bcl2 (Rat-Forward): 5′-cgggacgcgaagtgctattg-3′;

Bcl2 (Rat-Reverse): 5′-cggttgctctcaggctggaa-3′;

VEGFA (Rat-Forward): 5′-gagttaaacgaacgtacttgcaga-3′;

VEGFA (Rat-Reverse): 5′-tctagttcccgaaaccctga-3′;

VEGFA (Human-Forward): 5′-ttgctgctctacctccaccat-3′;

VEGFA (Human-Reverse): 5′-ggtgatgttggactcctcagtg-3′;

PTEN (Rat-Forward): 5′-ggcacaagaggccctggatt-3′;

PTEN (Rat-Reverse): 5′-tgcaagttccgccactgaaca-3′;

Jun (Rat-Forward): 5′-ccgtgagtgaccgcgacttt-3′;

Jun (Rat-Reverse): 5′-ctgggctgtgcgcagaagtt-3′;

Fos (Rat-Forward): 5′-accagagcgccccatcctta-3′;

Fos (Rat-Reverse): 5′-ctcctccgattccggcactt-3′;

APC2 (Rat-Forward): 5′-atcccaaggccacctggcta-3′;

APC2 (Rat-Reverse): 5′-tccccacaccgtcaccaagt-3′;

Ccna2 (Rat-Forward): 5′-ttgctggagctgccttccac-3′;

Ccna2 (Rat-Reverse): 5′-ctgttgggcatgctgtggtg-3′;

Cdc25a (Rat-Forward): 5′-ccttgccgatcgatgtggac-3′;

Cdc25a (Rat-Reverse): 5′-cgttggctccggaacatctg-3′;

Mcm3 (Rat-Forward): 5′-catccaggagatgccggaga-3′;

Mcm3 (Rat-Reverse): 5′-acgttgcaggcgatcaggac-3′;

Mcm6 (Rat-Forward): 5′-tgcaccaaccaacccacgat-3′;

Mcm6 (Rat-Reverse): 5′-gaacgccaccgaacagcatc-3′;

Ccnb2 (Rat-Forward): 5′-gctgggccaaggaaaatgga-3′;

Ccnb2 (Rat-Reverse): 5′-tgcctagggtctgcccatca-3′;

Mcm5 (Rat-Forward): 5′-tggcacagccaagtcacagc-3′;

Mcm5 (Rat-Reverse): 5′-cacggtcatcttcccgcatc-3′;

Cdk1 (Rat-Forward): 5′-cagatttcggccttgccaga-3′;

Cdk1 (Rat-Reverse): 5′-ttcttggtcgccagctctgc-3′;

Gadd45a (Rat-Forward): 5′-tgctcagcaaggctcggagt-3′;

Gadd45a (Rat-Reverse): 5′-gttgctgacccgcaggatgt-3′;

Myc (Rat-Forward): 5′-tcggctcccctgaaaagagc-3′;

Myc (Rat-Reverse): 5′-tcgctctgctgttgctggtg-3′;

Actin (Rat-Forward): 5′-cctctatgccaacacagtgc-3′;

Actin (Rat-Reverse): 5′-gtactcctgcttgctgatcc-3′

Actin (Human -Forward): 5′-gggacctgactgactacctc-3′;

Actin (Human -Reverse): 5′-tcatactcctgcttgctgat-3′

### Western Blot

Proteins were separated on a 12% SDS-PAGE gel and transferred to a nitrocellulose membrane (Bio-Rad, Hercules, USA). The membrane was blocked with 5% non-fat milk and incubated with Rabbit anti-VEGFA mAb (epitomics) or rabbit anti-Actin mAb (cell signaling). After being washed extensively, a goat anti-rabbit secondary antibody (Cell Signaling Technology, Massachusetts, USA) was added to the system. The proteins were detected using ECL reagents (Pierce).

### Enzyme-linked immunosorbent assays (ELISAs)

The level of VEGFA of supernatant from culture medium was measured using a commercially available ELISA kit, according to the manufacturer’s instructions (Excell biology, Shanghai, China). The minimal detectable concentration was 4 pg/mL for VEGFA. The inter-assay and intra-assay coefficients of variation were less than 10%.

### Gene silencing assay

MMQ tumor stem-like cells and MMQ cells grown in 6-well plates to ~50% confluency were serum-starved overnight and transfected with 100 pmol Silencer^®^ Select Negative Control #1 siRNA (Cat. No. 4390843, Ambion, USA) and Silencer^®^ Select Pre-Designed VEGFA siRNA (Cat. No. 4390771; ID: s236285, Ambion, USA) for 24 h using Lipofectamine™ 2000 (Invitrogen) according to the manufacturer’s instructions. After an additional 8 h incubation (serum-free medium), samples were collected at indicated times for further study.

### Cell proliferation assay

Cell proliferation was measured using the CCK-8 assay kit (Dojindo, Japan). 10,000 cells, treated with bevacizumab (Roche, 4 mg/μl) or DMSO for 72 h, were plated into each well of a 96-well plate. On the day of harvest, 10 μl CCK-8 was added to 90 μl of culture medium. The cells were subsequently incubated for 4 h at 37 °C and the optical density was measured at 450 nm. Three independent experiments were performed.

### Statistical analysis

Data were described as mean ± SEM. The *T* test statistic was applied to evaluate the difference between MMQ cells with tumor stem-like cells. *P* < 0.05 was considered as a significant difference. The calculations were performed with the software SPSS 17.0.

## Results

### Overview of gene expression array

**Fig. 1:**
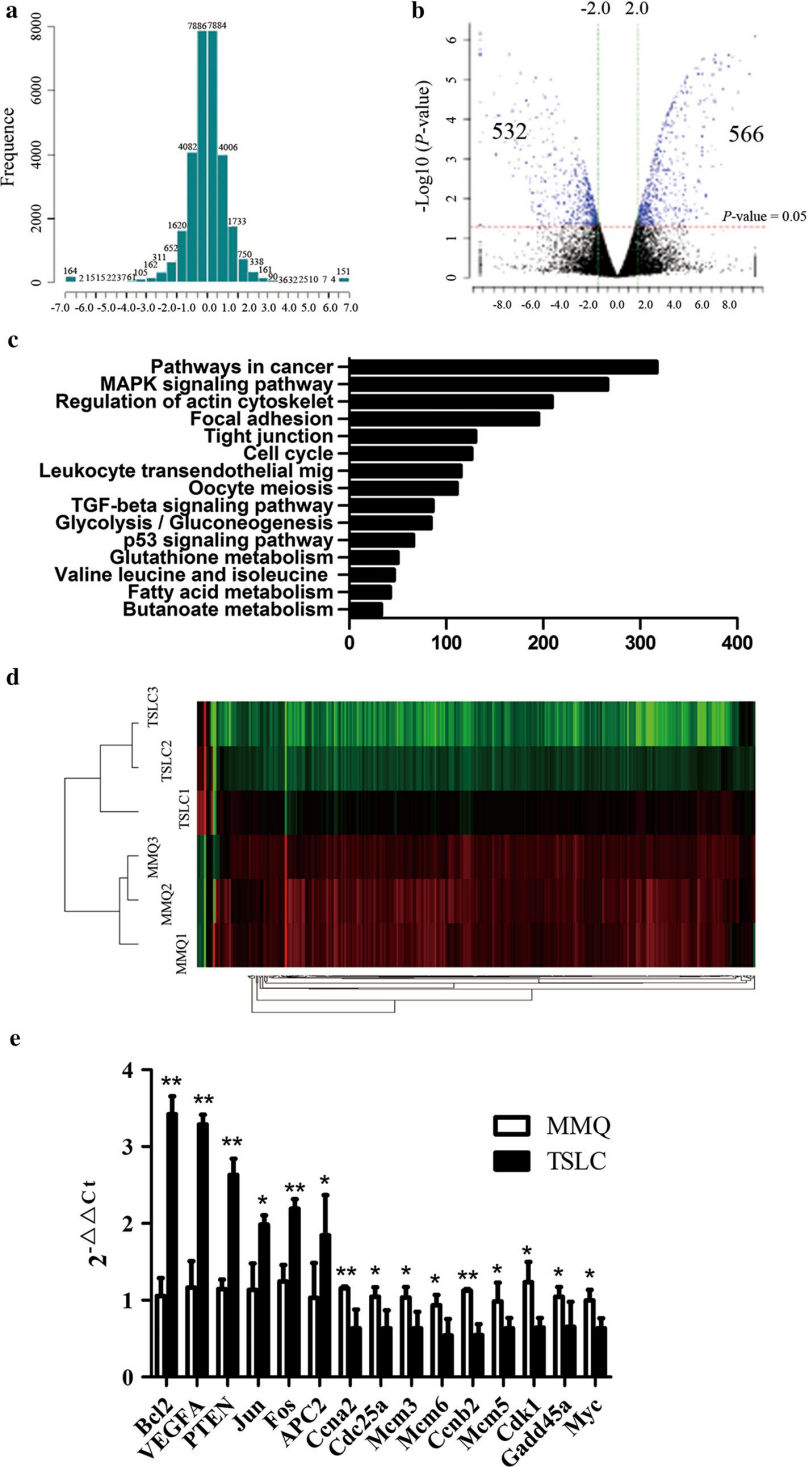
Overview of gene expression array. **a** Normal distribution of RNAs in classical MMQ cells and MMQ TSLCs. The Log_2_ absciss are presented the differences existing between the 2 group. The vertical axis showed the number of probe. In general, the figure become approximately normally distribution, which showed the up-regulated RNA should be roughly the same as the down-regulated RNA. **b** Volcano Plots of RNAs in classical MMQ cells and MMQ TSLCs. The Log_2_ absciss are presented the differences existing between the 2 group. The *vertical axis* showed the *P* value, which represented the significance of the difference. The *red line* mean the *P* = 0.05. The inclusion criteria of alternative gene: the expression of the gene was changed more than 2.0 folds (the *green line*) and the P value was less than 0.05 (the *red line*). Microarray showed that 566 known RNA were over-expressed and 532 known RNA were low-expressed in the MMQ TSLCs. **c** Biological processes analysis of the gene expression profiles. 15 pathway were shown to be significantly regulated with more than 500 genes differential expression in two group. **d** The cluster analysis of classical MMQ cells and MMQ TSLCs. Further study in pathway in cancer and cell cycle between MMQ cells and TSLCs were taken into consideration. According to the clustering analysis of three times independent microarray experiments, 82 candidate genes were selected. **E**. To verify the 82 candidate genes, MMQ cells and MMQ TSLCs were used to perform qRT-PCR for the identified RNAs. 15 differential expressed genes existed steady difference. *P < 0.05, **P < 0.01. *TSLC* tumor stem-like cell

Microarray systems were broadly used to investigate gene expression patterns and functional classification in stem cell [19]. In this paper, to evaluate the gene differences between MMQ cells and tumor stem-like cells, we extracted RNA from three classical cultured MMQ cells and three MMQ tumor stem-like cells cultured in serum-free suspension medium containing growth factor, and hybridized in Agilent Rat 8 × 60 K microarrays. The resulting data were analyzed by the GABRIEL (Genetic Analysis by Rules Incorporating Expert Logic) system, a knowledge based system of computer algorithms. As shown in Fig. 1a, the distributive differences of all detection gene probe (excluding controlled probe and flagged probe) in Agilent Rat 8 × 60 K microarrays tended to normal distribution, which was accurate and practical value. The expression levels of RNAs in the two libraries were normalized. The volcano plots of RNAs in classical MMQ cells and MMQ tumor stem-like cells was shown in Fig. 1b, the inclusion criteria of alternative gene: the expression of the gene was changed more than 2.0 folds and the *P* value was less than 0.05. After trimming sequences with contaminants, the results of Microarray showed that 566 known RNA were over-expressed (the blue spots on the right), and 532 known RNA were specially low-expressed in the MMQ tumor stem-like cells (the blue spots on the left).

### Biological processes analysis of the gene expression profiles

GO analysis is a functional analysis associating differentially expressed genes with GO categories. The GO categories are derived from Gene Ontology (http://www.geneontology.org), which comprises three structured networks of defined terms to describe gene product attributes. This functional analysis was used to predict significant differences between MMQ cells and tumor stem-like cells. As shown in Table 1, pathway in cancer, MAPK signaling pathway, regulation of actin cytoskeleton, focal adhesion, tight junction, cell cycle, leukocyte transendothelial migration, oocyte meiosis, TGF-beta signaling pathway, gluconeogenesis, P53 signaling pathway, glutathione metabolism, valine leucine and isoleucine degradation, fatty acid metabolism, butanoate metabolism were shown to be significantly regulated with more than 500 genes differential expression in two group (Fig. 1c; *P* value < 0.01).

**Table 1:** Biological processes analysis of the gene expression profiles

Geneset name	Genes in geneset	Description	Genes in overlap (k)	k/K	P value
Pathway in cancer	317	Genes annotated by the KEGG rno05200:pathways in cancer	25	0.07886	0.0159
MAPK signaling pathway	266	Genes annotated by the KEGG rno04010:MAPK signaling pathway	21	0.07895	0.0281
Regulation of action cytoskeleton	209	Genes annotated by the KEGG rno04810:regulation of action cytoskeleton	19	0.09091	0.0106
Focal adhesion	195	Genes annotated by the KEGG rno04510:focal adhesion	20	0.10256	0.0023
Tight junction	130	Genes annotated by the KEGG rno04530:tight junction	17	0.13077	0.0004
Cell cycle	126	Genes annotated by the KEGG rno04110:cell cycle	18	0.14286	8.7916E−05
Leukocyte transendothelial migration	115	Genes annotated by the KEGG rno04670:leukocyte transendothelial migration	14	0.12174	0.0031
Oocyt meiosis	111	Genes annotated by the KEGG rno04114:oocyt meiosis	12	0.10811	0.0163
TGF-beta signaling pathway	86	Genes annotated by the KEGG rno04350:TGF-beta signaling pathway	11	0.12791	0.0075
Glycolysis/gluconeogenesis	84	Genes annotated by the KEGG rno00010:glycolysis/gluconeogenesis	10	0.11905	0.018
p53 signaling pathway	66	Genes annotated by the KEGG rno04115:p53 signaling pathway	11	0.16667	0.001
Glutathione metabolism	50	Genes annotated by the KEGG rno00480:glutathione metabolism	7	0.14	0.0306
Valine leucine and isoleucine degradation	46	Genes annotated by the KEGG rno00280:valine leucine and isoleucine degradation	7	0.15217	0.0211
Fatty acid metabolism	42	Genes annotated by the KEGG rno00071:fatty acid metabolism	6	0.14286	0.0481
Butanoate metabolism	33	Genes annotated by the KEGG rno00650:butanoate metabolism	7	0.21212	0.0042

### Differential expression of RNAs between two groups in pathway in cancer and cell cycle

Earlier studies [20] had shown that cancer stem cells played a key role in neurogenic tumor regeneration, metastasis and recurrence. Cancer stem cells was resistant to conventional chemo- and radio-therapy, which was a important factor in drug resistance and recurrence [21–23]. Given that conventional therapies preferentially targeted cycling cells, quiescence was thought to render cancer stem cells resistant to such treatment [24, 25]. Based on the above, further study in pathway in cancer and cell cycle between MMQ cells and tumor stem-like cells were taken into consideration. According to the clustering analysis of three times independent microarray experiments (Fig. 1d). We rearranged the gene involved in these two cell signal pathway, stably changed more than 3.0-folds were chosen to in-depth study. As shown in Additional file 3: Table S1, 82 candidate genes were selected.

### Evaluation of gene expression changes by qRT-PCR

To verify the Microarray analysis results, ten classical cultured MMQ cells and ten MMQ tumor stem-like cells were used to perform qRT-PCR for the identified RNAs. 15 differential expressed genes existed steady difference, Bcl2, VEGFA, PTEN, Jun, Fos, APC2 were up-regulated and Ccna2, Cdc25a, Mcm3, Mcm6, Ccnb2, Mcm5, Cdk1, Gadd45a, Myc were down-regulated in MMQ tumor stem-like cells group (*P* < 0.05, Fig. 1e).

### Gene ontology (GO) category and pathway analysis

The cell signaling pathway of those 15 RNAs were predicted by KEGG and Database of gene ontology (GO). In pathway in cancer, we found that Bcl2, VEGFA, PTEN, Jun, Fos, APC2 gene expression were up-regulated and the expression of Myc was down-regulated in the MMQ TSLCs. In the pathway in cell cycle, we found that the expression of Ccna2, Cdc25a, Mcm3, Mcm6, Ccnb2, Mcm5, Cdk1, Gadd45a were down-regulated in the MMQ tumor stem-like cells. (see Additional file 4: Figure S3, Additional file 5: Figure S4)

### VEGFA expression was up-regulated in MMQ tumor stem-like cells

**Fig. 2:**
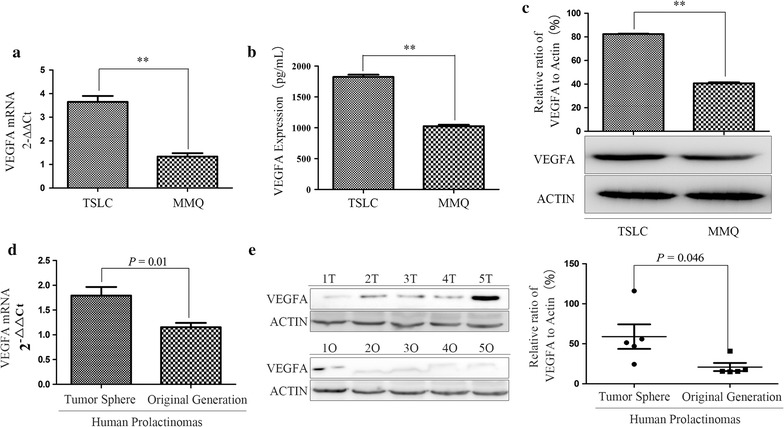
VEGFA expression was up-regulated in MMQ tumor stem-like cells and human prolactinoma tumor sphere cells. **a** VEGFA mRNA expression in MMQ TSLCs significantly increased compared with that in MMQ cells. **b** The VEGFA level of culture medium supernatant from MMQ TSLCs was higher than that from MMQ cells. **c** The protein expression of VEGFA significantly increased in MMQ TSLCs compared with MMQ cells evaluated by WB. **d** Human prolactinoma tumor spheres cultured in serum-free suspension medium had higher VEGFA mRNA, evaluated by qRT-PCR, compared with the original generation cells. **e** The expression of VEGFA protein of human prolactinoma tumor spheres was also significantly increased compared with that of the original generation cells. *TSLC* tumor stem-like cell, *T* human prolactinoma tumor spheres, *O* original generation cells of human prolactinoma. **P < 0.01

As previously reported [27], VEGFA was confirmed to associate with promoting cancer stemness, and played an important role in the neovascularization of TSLCs. To further investigate the exact effect of VEGFA in MMQ tumor stem-like cells. As shown in Fig. 2a, VEGFA mRNA expression in MMQ tumor stem-like cells were increased 273.2% compared with MMQ cells (*P* < 0.01). Morever, we found the level of VEGFA in culture medium supernatant of MMQ tumor sphere cells was 77.9% higher than that in MMQ cells (Fig. 2b, *P* < 0.01). The protein expression of VEGFA increased 102.3% in MMQ tumor sphere cells compared with MMQ cells (Fig. 2c, *P* < 0.01).

### VEGFA expression was up-regulated in human prolactinoma tumor sphere cells

In next set of experiments, we wanted to further validate the expression of VEGFA in human prolactinoma. Tumor cells from 5 human prolactinomas were collected and cultured in serum-free suspension medium for 2–3 weeks, which were similar to MMQ tumor stem-like cells, human prolactinoma tumor spheres could grow and form (see Additional file 2: Figure S2). Then we found that VEGFA mRNA increased 55.5% in human prolactinoma tumor spheres, and the expression of VEGFA protein was also significantly increased compared with the original generation cells (Fig. 2d, e).

### VEGFA silencing suppressed the growth of MMQ tumor stem-like cells in vitro

**Fig. 3:**
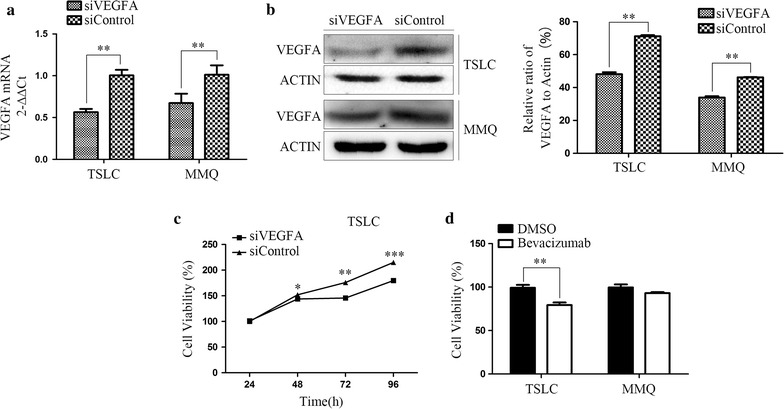
The growth-suppressive effect of VEGFA silencing and bevacizumab on MMQ tumor stem-like cells in vitro. **a**, **b** The effect of VEGFA knockdown via siRNA silencing in TSLC and MMQ cells. The cells were transfected with siControl or siVEGFA for 72 h and subjected to quantitative PCR and immunoblot analysis of VEGFA expression. **c** VEGFA knockdown via siRNA silencing inhibited the proliferation of TSLC. The cells were transfected with siControl or siVEGFA for 24–96 h and subjected to cell proliferation assay by CCK-8. **d** 10,000 MMQ TSLC, treated with bevacizumab (4 mg/μl) or DMSO for 72 h, were plated into each well of a 96-well plate and subjected to cell proliferation assay by CCK-8. The viability of MMQ TSLC treated with bevacizumab decreased compared with the negative DMSO group. TSLC: tumor stem-like cell. *P < 0.05, **P < 0.01, ***P < 0.001

To address the efficacy of VEGFA on MMQ tumor stem-like cells, we down-regulated the expression of VEGFA in MMQ cells and MMQ tumor stem-like cells by small interfering RNAs. As shown in Fig. 3a, b, MMQ cells and tumor stem-like cells transfected with VEGFA siRNA showed efficient silencing of VEGFA expression, as evaluaed by real-time RT-PCR (Fig. 3a) and immunoblot analysis (Fig. 3b). VEGFA mRNA expression decreased 43.7 and 33.9% in MMQ tumor stem-like cells and MMQ cells by siRNA silencing compared with siControl transfected cells, respectively. The protein expression of VEGFA decreased 32.4 and 26.6% in tumor stem-like cells and MMQ cells by siRNA silencing, respectively. In cell viability assay, tumor stem-like cells showed reduction of cell viability by VEGFA silencing compared with siControl transfected cells (Fig. 3c). At 96 h after transfected siRNA, cell viability reduced 16.5%. The cell viability has no difference between transfected with VEGFA siRNA and transfected with SiControl in MMQ cells (data not shown). These finding showed that VEGFA may be required for the growth of tumor stem-like cells in vitro.

### Bevacizumab could inhibit the proliferation of MMQ tumor stem-like cells in vitro

Bevacizumab, prevented solid tumor from growing by suppressing the production of VEGF, was the most commonly used anti-angiogenesis chemotherapy. The viability of MMQ tumor stem-like cells treated with bevacizumab decreased 19.7% compared with the DMSO group at 72 h. However, there were no significant difference in MMQ between bevacizumab group and DMSO group (Fig. 3d). These findings demonstrated that bevacizumab partially inhibited the proliferation of MMQ tumor stem-like cells.

## Discussion

An increasing body of evidence has indicated that cancer stem cells are the root of tumor metastasis and recurrence [26]. Cancer stem cells do not only reserve the characteristics of stem cells, but also have their own nature. The research of the tumor stem cells will give a new therapeutic approach for targeting tumor [27]. However, no study has been reported which investigated the RNAs expression profile for tumor stem-like cells isolated from pituitary prolactinoma.

The present study reports, to our knowledge for the first time, the RNA expression profile in MMQ tumor stem-like cells in comparison with MMQ cells. Agilent Rat 8 × 60 K Microarray was performed to screen the differential RNAs. The results of Microarray showed that 566 known RNA were over-expressed and 532 known RNA were low-expressed in the MMQ tumor stem-like cells. These genes were mainly involved in 15 different signaling pathways. After validated by qRT-PCR, we found that 15 RNAs were differentially expressed between MMQ tumor stem-like cells and MMQ cells in the pathway in cancer and cell cycle. Bcl2, VEGFA, PTEN, Jun, Fos, APC2 were up-regulated and Ccna2, Cdc25a, Mcm3, Mcm6, Ccnb2, Mcm5, Cdk1, Gadd45a, Myc were down-regulated in MMQ tumor stem-like cells group.

The theory of cancer stem cell proposed that the tumor tissue had its own cancer stem cells in it and regarded cancer stem cell as the key to regeneration, metastasis and recurrence [20]. If its stem cells were eradicated, the rest of a tumour might die off [28]. In order to clarify the differences of gene expression in tumour formation, we put research emphasis on the pathway in cancer. We found that Bcl2, VEGFA, PTEN, Jun, Fos, APC2 gene expression were up-regulated and the expression of Myc was down-regulated in the MMQ TSLCs. These differentially expressed genes were mainly involved in cell apoptosis and differentiation. Studies show that APC was an E3 ligase enzyme [29], which might combine with Bcl2 to protected TSLCs from apoptosis process by increasing the stability of mitochondrial outer membrane, halting the intrinsic apoptotic pathways [30] and possessing the ability to infinite differentiation. Jun and Fos were overexpression in MMQ tumor stem-like cells, they were the important component of AP-1 transcriptional activating complex [31], Which might play a key role in the differentiation of cancer stem cells. AP-1 could promote dopaminergic neuronal differentiation by combining with PTEN [32]. The expression alteration of these gene describe above suggested that MMQ tumor stem-like cells owned more efficient anti-apoptosis ability and were more tolerated than MMQ cells to the changes of cultured condition in vitro, such as medication treatment and nutrient scarcity. Their self-renewal and differentiation are under strict control, if the conditions are suitable, tumor stem-like cells could immediately differentiate into the daughter cells and form tumors rapidly. These results are consistent with previous studies [6, 7, 25, 33].

Earlier studies had shown that TSLCs maintained in a non-proliferative state (referred to as quiescence, dormancy, or G0 phase) and entered the cell cycle infrequently [33, 34]. Given that conventional therapies preferentially targeted cycling cells, quiescence was thought to render TSLCs resistant to chemo- and radio-therapy [24, 25]. In the pathway in cell cycle, we found that the expression of Ccna2, Cdc25a, Mcm3, Mcm6, Ccnb2, Mcm5, Cdk1, Gadd45a were down-regulated in the MMQ tumor stem-like cells. The expression of Ccna2, Ccnb2, and Cdk1 were low, which could reduce G1/S-phase arrest [35] and the transition from G2 to M phase [36], furthermore enhanced the ability of anti-apoptosis of tumor stem cells [37]. Mcm3, Mcm5, Mcm6 proteins had been identified as important participants of DNA replication [38] and Cdc25a [39] and Gadd45a [40] was essential for DNA repair. The low expression of the gene might contribute to the delay in cell cycle and the decrease of DNA repair, the damaged or mutated DNA might be a key role in tumor stem cells infinite proliferation. Our results might help to explain the mechanism of tumor stem-like cells maintained in a non-proliferative state.

One of the most interesting observations of the current study was that VEGFA had a distinct pattern of expression in MMQ tumor stem-like cells and was further validated in tumor spheres of human prolactinoma.

VEGFA was a central regulator of angiogenesis [41, 42]. VEGFA could created a perivascular niche for TSLCs by stimulating angiogenesis in a paracrine manner, and directly stimulated cancer stemness and renewal [43].

The pituitary contains abundant VEGFA [44], and VEGFA participated in the formation of the vascular network of a new pituitary tumor [45, 46], and increased tumoral VEGFA expression was observed during the development of estrogen-induced prolactinoma in rats [47]. These data indicated that VEGFA might contribute to adequate temporal vascular supply. Furthermore, VEGFA expression was related to invasion of pituitary adenoma [48]. The expression of VEGFA obviously increased in pituitary adenoma with extrasellar growth than that in intrasellar ones, suggesting VEGFA could be markers for poor outcome after tumor resection [49]. Previous studies [50, 51] revealed that VEGFA protein expression was high in the dopamine agonist resistant prolactinomas. Furthermore, the patients with tumor recurrence showed a significant rise in serum concentrations of VEGFA. The antiangiogenic treatments were effective in inhibiting the growth of primary dopamine resistant prolactinomas as well as the transplanted adenomas [52]. In our study, both expression and secretion of VEGFA increased obviously in tumor stem-like cells of prolactinomas. Our results, integrating with the reports mentioned above, indicated that VEGFA secreted by tumor stem-like cells might play a key role in the occurrence and progression of prolactinoma and act as a potential therapeutic target.

There are several limitations in this study. Firstly, the high expression of VEGFA in MMQ tumor stem-like cells is tested in vitro, the in vivo environment is so much more complicated than in cell lines. Secondly, the regulation mechanisms of VEGFA in the tumor stem-like cells is not clear. Here, a further experiment is needed to confirm how the VEGF is involved in human prolactinomas formation and progression in vivo. This may help us to have a much better understanding of the mechanism for the beneficial effect of tumor stem-like cells on prolactinomas formation.

## Conclusion

In conclusion, our study characterize the pattern of RNA expression of MMQ stem-like cells. This finding may provide potential targets for development of TSLC-based therapies and lay the foundation for the further study in RNA-involved mechanism of MMQ tumor stem-like cells in the future.

## Additional files

**Additional file 1: Figure S1.** The characteristic identification of MMQ TSLC.

**Additional file 2: Figure S2.** The characteristic identification of tumor spheres isolated from human prolactinoma.

**Additional file 3: Table S1.** 82 candidate genes in pathway in cancer and cell cycle between MMQ TSLCs and MMQ cells.

**Additional file 4: Figure S3.** GO analysis and KEGG pathway analysis of 15 differentially expressed genes for the pathway in cancer.

**Additional file 5: Figure S4.** GO analysis and KEGG pathway analysis of 15 differentially expressed genes for the pathway in cell cycle.

## Abbreviations

Actin: alpha 1, skeletal muscle
APC2: adenomatosis polyposis coli 2
Bcl2: B-Cell CLL/lymphoma 2
bFGF: basic fibroblast growth factor
CCK-8: cell counting kit-8
Ccna2: cyclin A2
Ccnb2: cyclin B2
Cdc25a: cell division cycle 25A
Cdk1: cyclin-dependent kinase 1
CSCs: cancer stem cells
Ct: cycle threshold
DA: dopamine agonist
DAVID: database for annotation, visualization and integrated discovery
DEPC: diethy pyrocarbonate
DMSO: dimethyl sulfoxide
EGF: epidermal growth factor
ELISA: enzyme linked immunosorbent assay
Fos: FBJ murine osteosarcoma viral oncogene homolog
Gadd45a: growth arrest and DNA damage inducible alpha
GO: gene ontology
Jun: Jun Proto-Oncogene
KEGG: kyoto encyclopedia of genes and genomes
Mcm3: minichromosome maintenance complex component 3
Mcm5: minichromosome maintenance complex component 5
Mcm6: minichromosome maintenance complex component 6
MMQ: MMQ rat pituitary prolactinoma cell
Myc: V-Myc avian myelocytomatosis viral oncogene homolog
PTEN: phosphatase and tensin homolog
qRT-PCR: quantitative real time polymerase chain reaction
SEM: standard error of mean
TSLCs: tumor stem-like cells
VEGFA: vascular endothelial growth factor A
WB: western blot
