# Supplementary material for: Global expression profile of tumor stem-like cells isolated from MMQ rat prolactinoma cell
Source: Cancer Cell Int. 2017 Jan 31;17:15. doi: 10.1186/s12935-017-0390-1 (PMC5282624; doi:10.1186/s12935-017-0390-1)
Supplement: Supplementary file 1 — Additional file 1: Figure S1. The characteristic identification of MMQ TSLC. [file 12935_2017_390_MOESM1_ESM.docx]

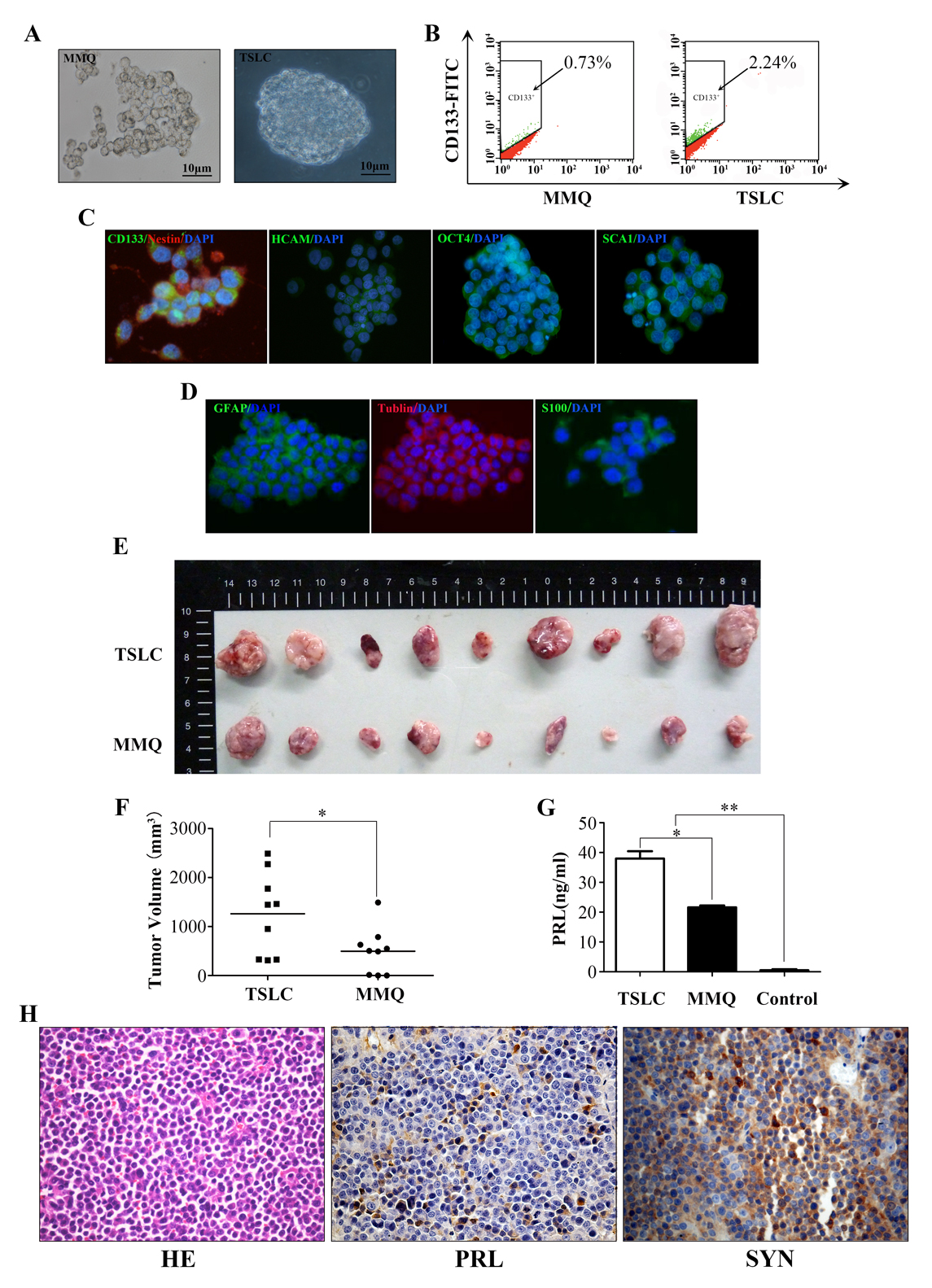


**Supplement Figure 1** **The characteristic identification of MMQ TSLC.** **A**. MMQ cells were cultured in serum-free suspension medium. After been cultured for 2 to 3 weeks, MMQ TSLC can grow and form. **B**. The expression of CD133 in MMQ cells and MMQ TSLC were identified by FACS. The result showed the ratio of CD133-positive cells in MMQ TSLC was 2.24%, higher than that was in MMQ cells, of which the ratio was 0.73%. **C**. The expression of stem cell markers in MMQ TSLC was detected by immunofluorescence. Co-expressing of CD133 (green) and Nestin (red) were presented in TSLC. In addition, a small fraction of MMQ TSLC was detected the positive expression of HCAM (green), OCT4 (green) and SCA1 (green), which were also considered as markers of stem cells. **D**. After differentiation culture of MMQ TSLC, phenotype differentiation and surface marker of GFAP (green), Tublin (red) and S100 (green) were expressed in the cells. **E and F**. MMQ cells (1×10^6^) were subcutaneously injected into flanks of each nude mice (n=9) and TSLC group (n=9) underwent the same way of injection with MMQ TSLC (1×10^5^) simultaneously. After injection, tumor size was measured by a vernier caliper weekly and calculated as (length×width^2^)/2. When the tumors increased to about 10 mm in diameter, the MMQ group nude mice (n=9) and MMQ TSLC group nude mice (n=9) were euthanized, xenograft tumors were harvested and blood was collected from the angular vein. The representative images for xenograft tumor on the nude mouse are shown in **E** and the tumor volume is shown in **F**. **G**. The serum PRL levels of MMQ group and MMQ TSLC group were determined using a rat PRL ELISA kit, the expression of PRL in TSLC group was significantly higher. **H**. A HE Staining Kit was used to characterize the histological features of TSLC group (left). The PRL (middle) and SYN (right) expression of tumor tissues was detected according to the standard protocol of immunohistochemistry. TSLC: tumor stem-like cell. * *P* < 0.05, ** *P* < 0.01.
