# Supplementary material for: Global expression profile of tumor stem-like cells isolated from MMQ rat prolactinoma cell
Source: Cancer Cell Int. 2017 Jan 31;17:15. doi: 10.1186/s12935-017-0390-1 (PMC5282624; doi:10.1186/s12935-017-0390-1)
Supplement: Supplementary file 2 — Additional file 2: Figure S2. The characteristic identification of tumor spheres isolated from human prolactinoma. [file 12935_2017_390_MOESM2_ESM.docx]

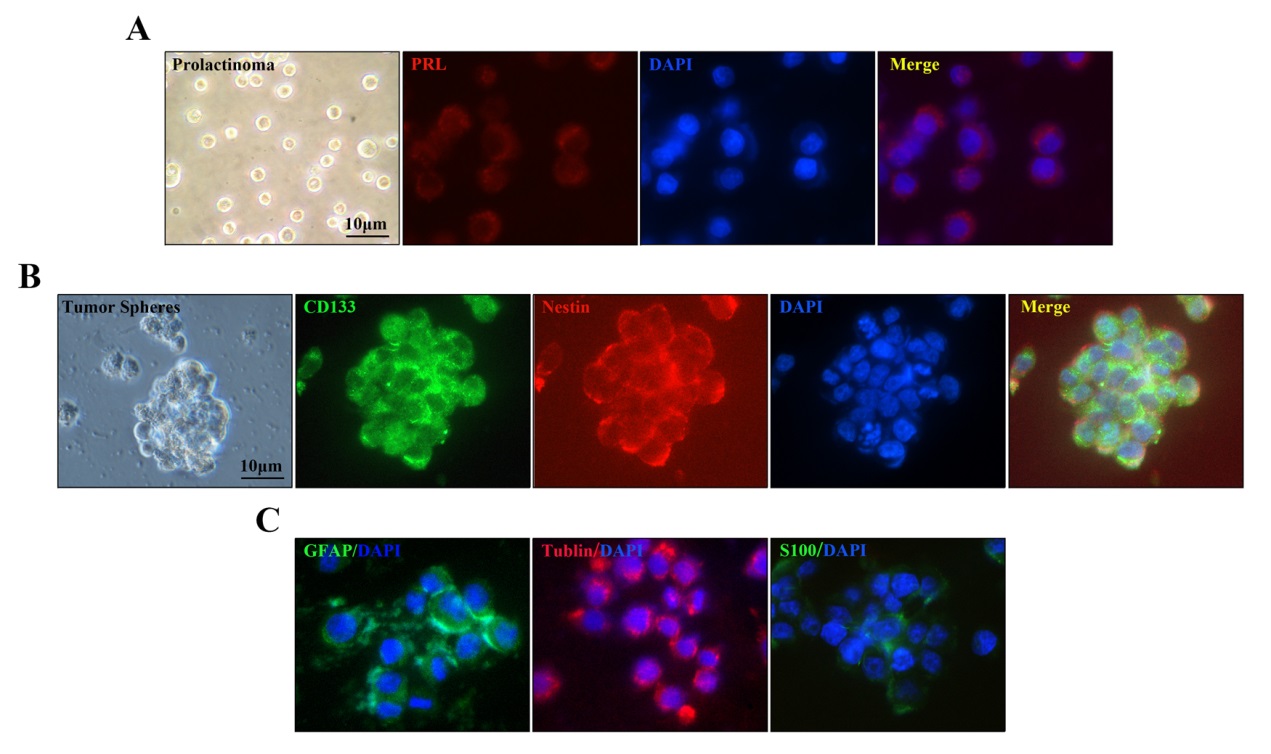


**Supplement Figure 2 The characteristic identification of tumor spheres isolated from human prolactinoma.** **A**. Human prolactinoma tumor cells were suspension cultured in DMEM medium and the expression of PRL was detected by immunofluorescence. **B**. Prolactinoma tumor cells were collected and cultured in serum-free suspension medium. After been cultured for 2 to 3 weeks, human prolactinoma tumor spheres can grow and form. The expression of stem cell markers CD133 (green) and Nestin (red) in prolactinoma tumor spheres cells was detected. **C**. After differentiation culture of human prolactinoma tumor spheres cells, phenotype differentiation and surface marker of GFAP (green), Tublin (red) and S100 (green) were expressed in the cells.
