# Supplementary material for: Global expression profile of tumor stem-like cells isolated from MMQ rat prolactinoma cell
Source: Cancer Cell Int. 2017 Jan 31;17:15. doi: 10.1186/s12935-017-0390-1 (PMC5282624; doi:10.1186/s12935-017-0390-1)
Supplement: Supplementary file 3 — Additional file 3: Table S1. 82 candidate genes in pathway in cancer and cell cycle between MMQ TSLCs and MMQ cells. [file 12935_2017_390_MOESM3_ESM.docx]

**Supplement Table 1.** 82 candidate genes in Pathway in Cancer and Cell Cycle between MMQ TSLCs and MMQ cells.
