# Supplementary material for: Global expression profile of tumor stem-like cells isolated from MMQ rat prolactinoma cell
Source: Cancer Cell Int. 2017 Jan 31;17:15. doi: 10.1186/s12935-017-0390-1 (PMC5282624; doi:10.1186/s12935-017-0390-1)
Supplement: Supplementary file 4 — Additional file 4: Figure S3. GO analysis and KEGG pathway analysis of 15 differentially expressed genes for the pathway in cancer. [file 12935_2017_390_MOESM4_ESM.docx]

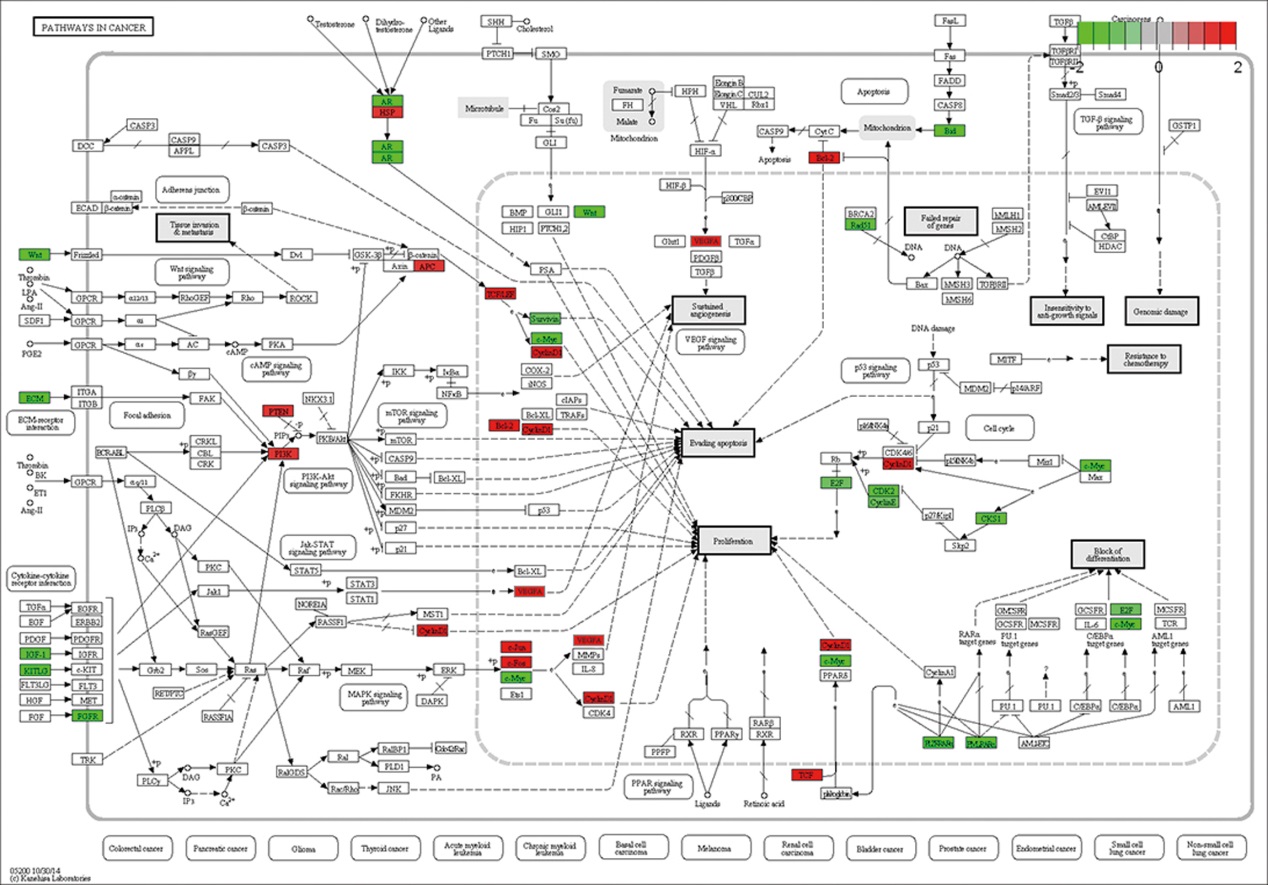


**Supplement Figure 3 GO analysis and KEGG pathway analysis of 15 significantly differential expression RNA were performed by DAVID Bioinformatics Resources**. GO category for the Pathway in Cancer, Bcl2, VEGFA, PTEN, Jun, Fos, APC2 gene expression were up-regulated and the expression of Myc was down-regulated in the MMQ tumor stem-like cells.
