# Supplementary material for: Global expression profile of tumor stem-like cells isolated from MMQ rat prolactinoma cell
Source: Cancer Cell Int. 2017 Jan 31;17:15. doi: 10.1186/s12935-017-0390-1 (PMC5282624; doi:10.1186/s12935-017-0390-1)
Supplement: Supplementary file 5 — Additional file 5: Figure S4. GO analysis and KEGG pathway analysis of 15 differentially expressed genes for the pathway in cell cycle. [file 12935_2017_390_MOESM5_ESM.docx]

**

**

**Supplement Figure 4 GO analysis and KEGG pathway analysis of 15 significantly differential expression RNA were performed by DAVID Bioinformatics Resources**. GO category for the Cell Cycle, Ccna2, Cdc25a, Mcm3, Mcm6, Ccnb2, Mcm5, Cdk1, Gadd45a were down-regulated in MMQ tumor stem-like cells group.
